# Prevalence of tuberculosis among prisoners in sub-Saharan Africa: a systematic review and meta-analysis

**DOI:** 10.3389/fpubh.2023.1235180

**Published:** 2023-12-28

**Authors:** Yordanos Sisay Asgedom, Gizachew Ambaw Kassie, Tsegaye Melaku Kebede

**Affiliations:** ^1^Department of Epidemiology, Wolaita Sodo University, Sodo, Ethiopia; ^2^Institute of Health, Jimma University, Oromia, Ethiopia

**Keywords:** tuberculosis, prison, meta-analysis, sub-Saharan Africa, systematic review

## Abstract

**Background:**

Tuberculosis (TB) is a key community health problem in numerous settings, predominantly in sub-Saharan Africa (SSA). TB is the second most lethal infectious disease worldwide. Around 1.6 million people died from TB in 2021. TB prevention and control strategies are difficult to implement in prison, especially in sub-Saharan Africa, owing to overcrowding and poor ventilation. Thus, this systematic review and meta-analysis aimed to synthesize the estimated pooled prevalence of tuberculosis among prisoners in sub-Saharan Africa.

**Materials and methods:**

Electronic biomedical databases such as Google Scholar, Web of Science, PubMed/Medline, EMBASE, and Science Direct were used to systematically explore candidate studies published until December 2022. Data extraction was performed using a Microsoft Excel spreadsheet. The estimated pooled prevalence of tuberculosis was determined using a fixed-effects model. Cochrane Q-test and I^2^ statistics were used to check heterogeneity statistically across different studies. Begg’s rank and Egger’s tests were performed to assess evidence of possible publication bias.

**Results:**

A total of 40 articles involving 59,300 prisoners were included in this systematic review and meta-analysis. The pooled prevalence of tuberculosis was 4.02% (95% CI: 2.68–5.36). We found the highest prevalence using Gene X pert as a diagnostic method, which was 4.97 (95% CI: 2.22–7.73). There is no evidence of publication bias.

**Conclusion:**

The outcome of this review revealed a high prevalence of tuberculosis among prisoners in sub-Saharan Africa. To reach the “End Tuberculosis strategy” by 2030, early identification of cases through screening on entry and periodical active case finding is important. Moreover, prevention and prompt treatment after diagnosis must be implemented to limit transmission to the general population.

**Systematic review registration:**

https://www.crd.york.ac.uk/prospero/#searchadvanced, identifier (CRD42023428933).

## Introduction

*Mycobacterium tuberculosis* complex (MTBC) causes tuberculosis. When a person coughs, sneezes, talks, or sings, droplet nuclei are produced, which spread from person to person through the air (1, 2). Coughing for more than 2 weeks, fever, weight loss, and sputum production can occur in conjunction with hemoptysis, loss of appetite, night sweats, and fatigue, which are expressive clinical signs in patients positive for pulmonary tuberculosis (3).

In 2021, approximately 1.6 million people died and 10.6 million people contracted tuberculosis (TB) worldwide. Low-and middle-income countries accounted for 80% of cases and deaths, with 23% of new cases in the World Health Organization (WHO) Africa region. TB is the second biggest killer among infectious diseases and the 13th leading cause of death worldwide (1).

Globally, there were approximately 11 million people imprisoned in 2018. An increment of approximately 24% was observed between 2000 and 2018 globally. The imprisoned population in Africa has increased by 29% in recent years, and the tuberculosis burden in this region is the highest compared to other WHO regions (1). The prison system is a potential area for transmitting communicable diseases such as tuberculosis due to overcrowding, poor ventilation, inadequate lighting, illicit drug use, difficulty accessing health services, lack of or precarious basic sanitation housing infrastructure, and malnutrition (1, 3–5).

In developing countries, TB is more common in prisons than in the general population, and prisons in SSA are riskier due to the high number of incarcerated people per cell block, ventilation systems, nutrition-related issues, and high prevalence of human immunodeficiency virus (HIV) (6–9). TB prevalence among prisoners from 24 SSA countries ranges from 0.4 to 16.3% (10).

Prison staff are at risk of contracting tuberculosis due to their interaction with their inmates, which leads to the spread of the disease to their families and communities. This suggests that tuberculosis in prison is a concern for society as a whole, not just for prisoners (7). Therefore, compared to other regions in the world, SSA is one of the regions with a high burden of TB; therefore, this systematic review and meta-analysis helps to update the prevalence of tuberculosis among prisoners, inform policymakers, and improve approaches to prisoners.

## Materials and methods

### Reporting

The results were reported using the Preferred Reporting Items for Systematic Reviews and Meta-Analyzes (PRISMA) statement (11). The article screening was based on the PRISMA 2009 statement, and the selection process has been shown using a PRISMA-P flow diagram. This review is registered in PROSPERO with registration number CRD42023428933.

### Search methods and strategies

To identify potentially relevant articles, we performed exhaustive searches of electronic databases with no date limits on Google Scholar, Web of Science, PubMed/MEDLINE, Science Direct, PubMed/MEDLINE, and EMBASE. All searches were limited to articles written in English and human studies. We conducted a manual search for additional relevant studies using references from retrieved articles and related systematic reviews to identify original articles that may have been overlooked. The following keywords were used to generate search strings or terms: prevalence, magnitude, Tuberculosis, Pulmonary Tuberculosis, Mycobacterium infections, Prisoners, and sub-Saharan Africa. Advanced search databases were built with the above-mentioned terms in mind, using “Medical Subject Headings (MeSH) [((((“Prevalence”) OR “Burden” OR “Magnitude”) AND “tuberculosis” AND “prisoners”)) AND sub-Saharan Africa].

### Inclusion and exclusion criteria

All studies on the prevalence of tuberculosis among prisoners in sub-Saharan Africa were included. Furthermore, this systematic review and meta-analysis included all cross-sectional studies on prisoners published in English and conducted in sub-Saharan Africa. Review papers, case series, case reports, abstracts, and qualitative studies were also barred from consideration.

### Outcome measurement

One major finding that emerged from this systematic review and meta-analysis is the estimation of the pooled prevalence of tuberculosis among prisoners in sub-Saharan Africa. A tuberculosis-positive patient has a *Mycobacterium tuberculosis* complex found in a clinical specimen, whether by smear, culture, or WHO-recommended rapid diagnosis (such as Xpert MTB/RIF).

### Data extraction and quality assessment

Endnote citation manager software version X9 for Windows was used to import retrieved studies from the databases, and manual removal was performed for duplicated articles. All articles were screened by three independent reviewers for predefined inclusion and exclusion criteria (abstract and title), followed by a full-text review. If disagreements regarding the inclusion of studies could not be resolved, a fourth investigator was invited to reach an agreement. Excel spreadsheet software was used to extract the data from the included studies. The spreadsheet included the first author’s name, publication year, study design, country, sample size, diagnostic methods, and number of cases (Table 1).

**Table 1:** The baseline characteristics of the included studies, 2023.

Authors	Year	Country	Sample size	Diagnostic methods	No_of cases	Prevalence (%)
Abebe et al. (12)	2011	Ethiopia	382	Sputum smear microscopy	33	8.9
Adane et al. (13)	2019	Ethiopia	1,124	Sputum smear microscopy	34	3
Adane et al. (14)	2016	Ethiopia	809	Sputum smear microscopy	74	5.88
Addis et al. (15)	2015	Ethiopia	384	Sputum smear microscopy	33	8.59
Adesokan et al. (16)	2014	Nigeria	164	Culture	2	1.2
Agajie et al. (17)	2018	Ethiopia	84	GeneXpert	8	9.5
Ali et al. (18)	2015	Ethiopia	765	Sputum smear microscopy	71	9.2
Banda et al. (19)	2009	Malawi	7,661	Sputum smear microscopy	54	0.7
Bayu et al. (20)	2016	Ethiopia	302	Sputum smear microscopy	17	5.57
Berihun et al. (21)	2018	Ethiopia	162	Sputum smear microscopy	32	19.6
Biyadgilign et al. (22)	2014	Ethiopia	200	GeneXpert	16	8
Chigbuand Iroegbu (23)	2010	Nigeria	168	Interferon Gamma Release Assay	22	13
Chekesa et al. (24)	2020	Ethiopia	352	Sputum smear microscopy	180	51.2
Dibissa et al. (25)	2019	Ethiopia	249	GeneXpert	15	6
Fuge et al. (26)	2016	Ethiopia	164	Sputum smear microscopy	3	1.8
Gebrecherkos et al. (27)	2016	Ethiopia	282	GeneXpert	15	5.3
Gizachew et al. (28)	2017	Ethiopia	265	GeneXpert	9	3.4
Habeenzu et al. (29)	2007	Zambian	1,080	Culture	245	22.7
Henostriza et al. (30)	2013	Zambian	2,323	Sputum smear microscopy	88	3.8
Jordan et al. (31)	2019	South Africa	31,843	GeneXpert	859	2.7
Kalonji et al. (32)	2016	DRC	733	Sputum smear microscopy	130	17.7
Kanyerere et al. (33)	2012	Malawi	2,217	Sputum smear microscopy	44	2
Kwabla et al. (34)	2015	Ghana	151	GeneXpert	1	0.9
Keyomo et al. (35)	2018	DRC	918	Sputum smear microscopy	27	2.9
Lawal et al. (36)	2009	Nigerian	2002	Sputum smear microscopy	48	2.4
Maggard et al. (37)	2015	Zambian	7,638	Culture	306	4
Merid et al. (38)	2018	Ethiopia	372	GeneXpert	34	9.13
Mohammed et al. (39)	2017	Ethiopia	765	GeneXpert	23	3
Moges et al. (40)	2012	Ethiopia	250	Sputum smear microscopy	26	10.4
Mmbaga et al. (41)	2013	Tanzania	448	Sputum smear microscopy	22	5
Mpeirwe et al. (42)	2016	Uganda	140	Sputum smear microscopy	11	8
Noeske et al. (43)	2006	Cameroon	2,474	Sputum smear microscopy	87	3.5
Nyangulu et al. (44)	1997	Malawi	914	Sputum smear microscopy	47	5.1
Owokuhaisa et al. (45)	2014	Uganda	248	Sputum smear microscopy	2	1.2
Sesay et al. (46)	2016	Ghana	148	GeneXpert	12	8
Seri et al. (47)	2017	Cote d’ivoire	943	Sputum smear microscopy	89	9.3
Telisinghe et al. (48)	2014	South Africa	981	Sputum smear microscopy	34	3.4
Winsa et al. (49)	2015	Ethiopia	196	Sputum smear microscopy	43	21.9
Zerdo et al. (50)	2014	Ethiopia	3,817	GeneXpert	24	19.35
Zishiri et al. (51)	2015	South Africa	4,945	GeneXpert	445	9

### Statistical analysis

The analysis was carried out using the statistical software STATA Version 14.1 (StataCorp, College Station, Texas, United States), and heterogeneity was checked across studies by computing the I2 statistical test. If the I^2^ values were 0, 25, 50, and 75%, we assumed no, low, medium, and high heterogeneity across studies. A meta-analysis using a fixed-effects model with 95% confidence intervals (CI) was performed to analyze the pooled prevalence of tuberculosis among prisoners (I^2^,16.3% *p* = 0.188). A visual inspection of the funnel plot was performed to check for evidence of publication bias, followed by Begg’s rank and Egger’s tests, with a value of p of less than 0.05 used as a cut-off point. Leave-one-out sensitivity analysis was also performed to assess the impact of a small study. The analysis was carried out step-by-step, excluding the study, to assess the effect of each study on the pooled prevalence of tuberculosis. A forest plot was used to estimate pooled prevalence.

## Results

### Selection of studies

**Figure 1:**
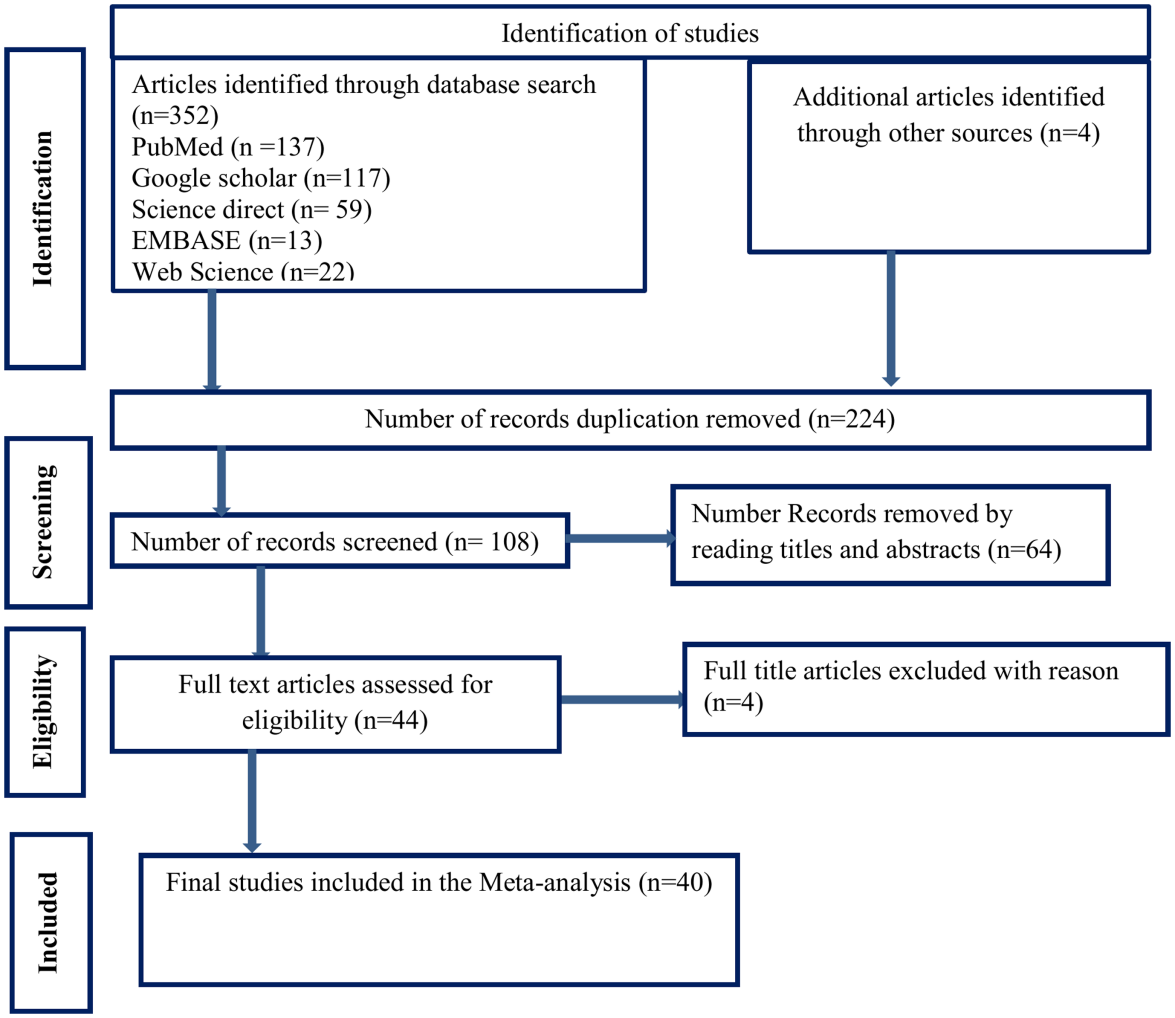
PRISMA flow diagram of articles screened and the selection process on tuberculosis among prisons in sub-Saharan, 2023.

A search of the biomedical electronic databases yielded 352 published and unpublished studies. Although 244 duplicate articles were identified and removed, 108 were included in the screening. After 64 studies were removed based on title and abstract screening, 44 studies remained. Finally, 40 studies that met the eligibility criteria were included in the final analysis to estimate the pooled prevalence of tuberculosis among sub-Saharan African prisoners. The full selection process is illustrated in Figure 1.

### Included studies characteristics

Among the 40 included studies, there were 19 studies were from Ethiopia; 3 from South Africa; 3 from Nigeria, Malawi, and Zambia; 2 from the Democratic Republic of Congo (DRC), Uganda, and Ghana; and 1 from Cameroon, Tanzania, and Cote d’Ivoire. The included study sample size ranged from 84 (2) to 31,843 (3), with 80,608 prisoners. Observational and interventional studies published between 1997 and 2020 were included. All 40 articles had a cross-sectional design. All included studies were facility-based (Table 1 illustrates the included studies’ baseline characteristics).

### The pooled prevalence of tuberculosis among prisoners in sub-Saharan Africa

**Figure 2:**
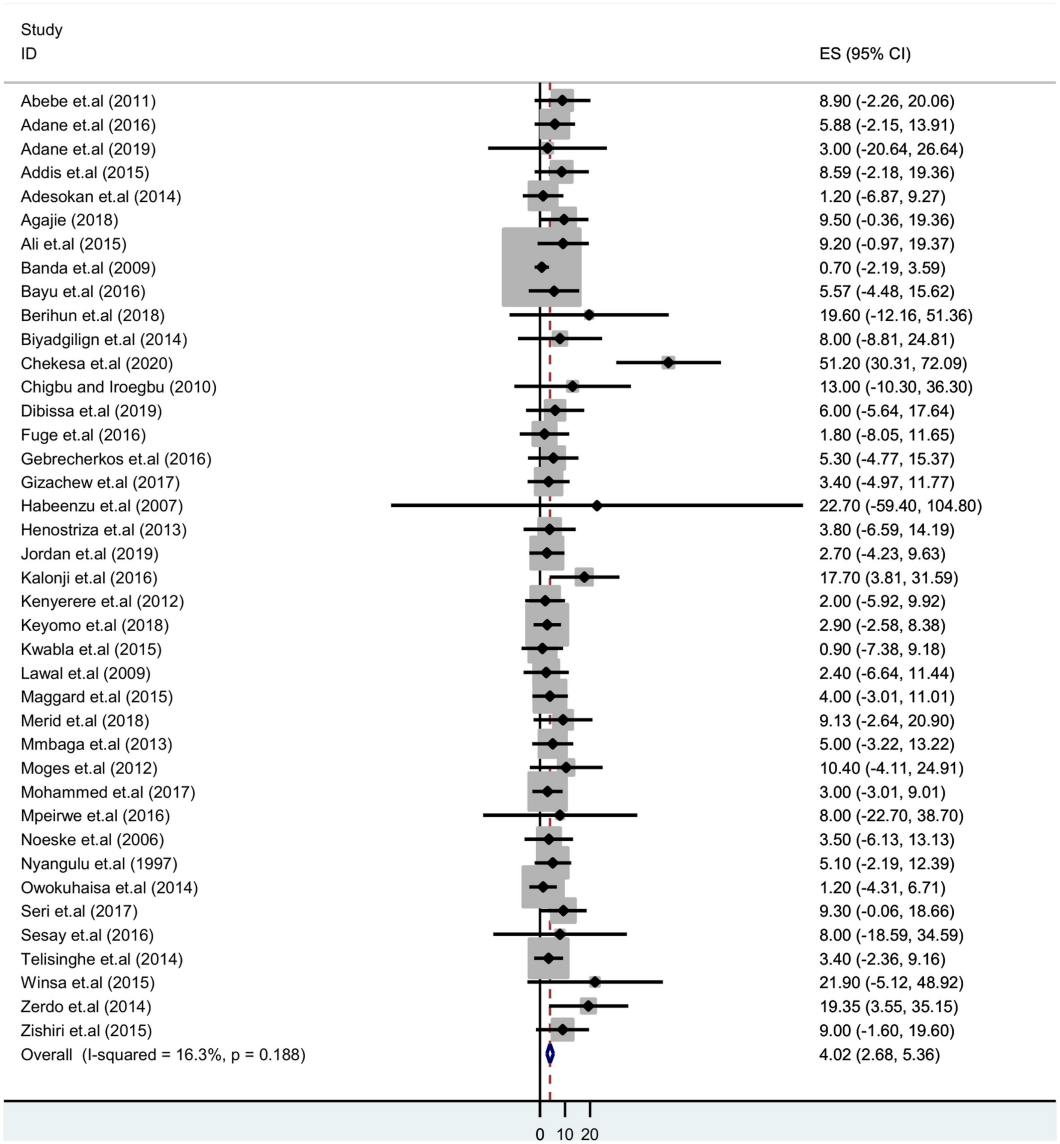
Forest plot showing pooled prevalence estimate of tuberculosis infection among prisoners in sub-Saharan Africa, 2023.

The pooled prevalence of tuberculosis among sub-Saharan African prisoners was 4.02% (95% CI: 2.68–5.36). The forest plot shows that statistical heterogeneity was low (I^2^ = 16.3%; *p* 0.188). As a result, we used a fixed effects model to estimate the pooled prevalence of tuberculosis (Figure 2).

### Sub-group analysis

**Figure 3:**
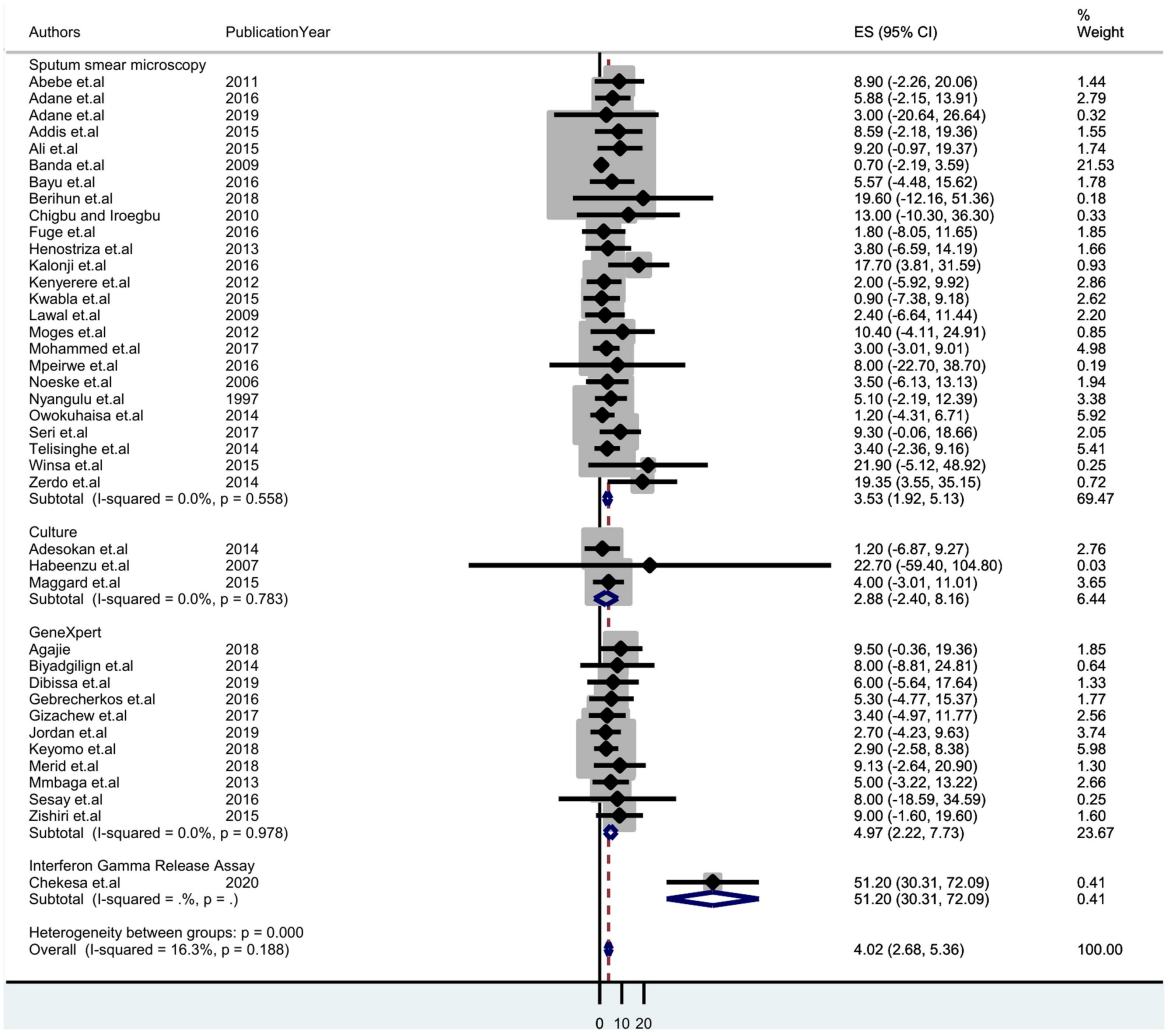
Forest plot displaying subgroup analysis on the pooled prevalence of tuberculosis by diagnostic method among prisoners in sub-Saharan Africa, 2023.

**Figure 4:**
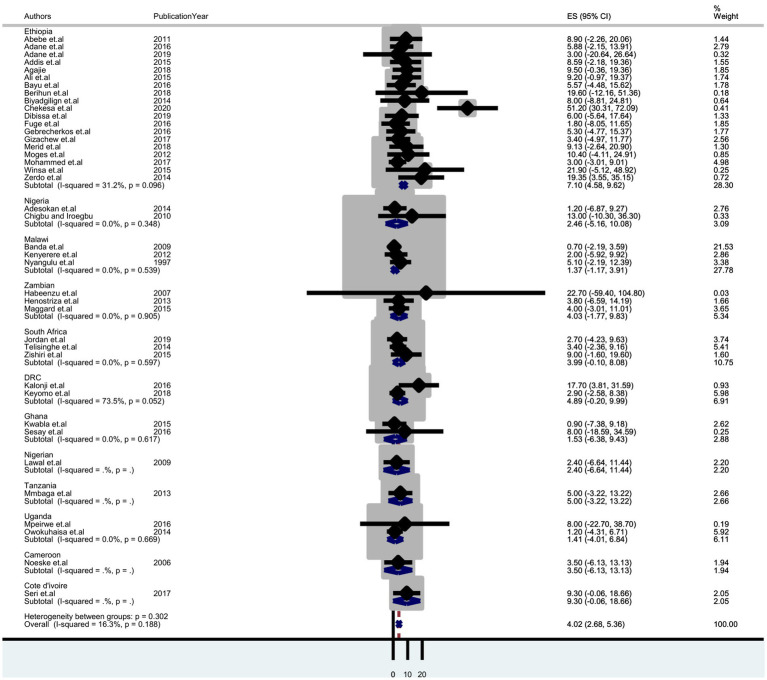
Forest plot displaying subgroup analysis on the pooled prevalence of tuberculosis by country among prisoners in sub-Saharan Africa, 2023.

A sub-group analysis based on diagnostic methods and country setting was performed to identify potential sources of heterogeneity. It shows the highest detection of tuberculosis was by Gene Xpert, which was 4.97% (95% CI: 2.22–7.73); sputum smear microscopy was 3.53% (95% CI: 1.92–5.13) and culture was 2.88% (95% CI: 2.40–8.16) (Figure 3). Thus, we observed country variation in the prevalence of tuberculosis in this study. The prevalence of TB was found to range between 7.10 (95% CI: 4.58–9.62) in Ethiopia and 1.37 (95% CI:-1.17–3.91) in Malawi (Figure 4).

### Meta-regression

Meta-regression was used to identify factors associated with the pooled prevalence of tuberculosis among prisoners while keeping continuous variables in mind. For the meta-regression, publication year and sample size were considered. Meta-regression analysis revealed no statistically significant relationship between the pooled prevalence of tuberculosis among prisoners and publication year or sample size (Table 2).

**Table 2:** Meta-regression to identify the source of heterogeneity for the pooled prevalence of tuberculosis among prisoners in sub-Saharan Africa, 2023.

Prevalence	Coefficient	[95% Conf. Interval]	*p*-value
Publication year	0.227	(−0.120—0.575)	0.194
Sample size	0.038	(−0.120—0.197)	0.626

### Publication bias

**Figure 5:**
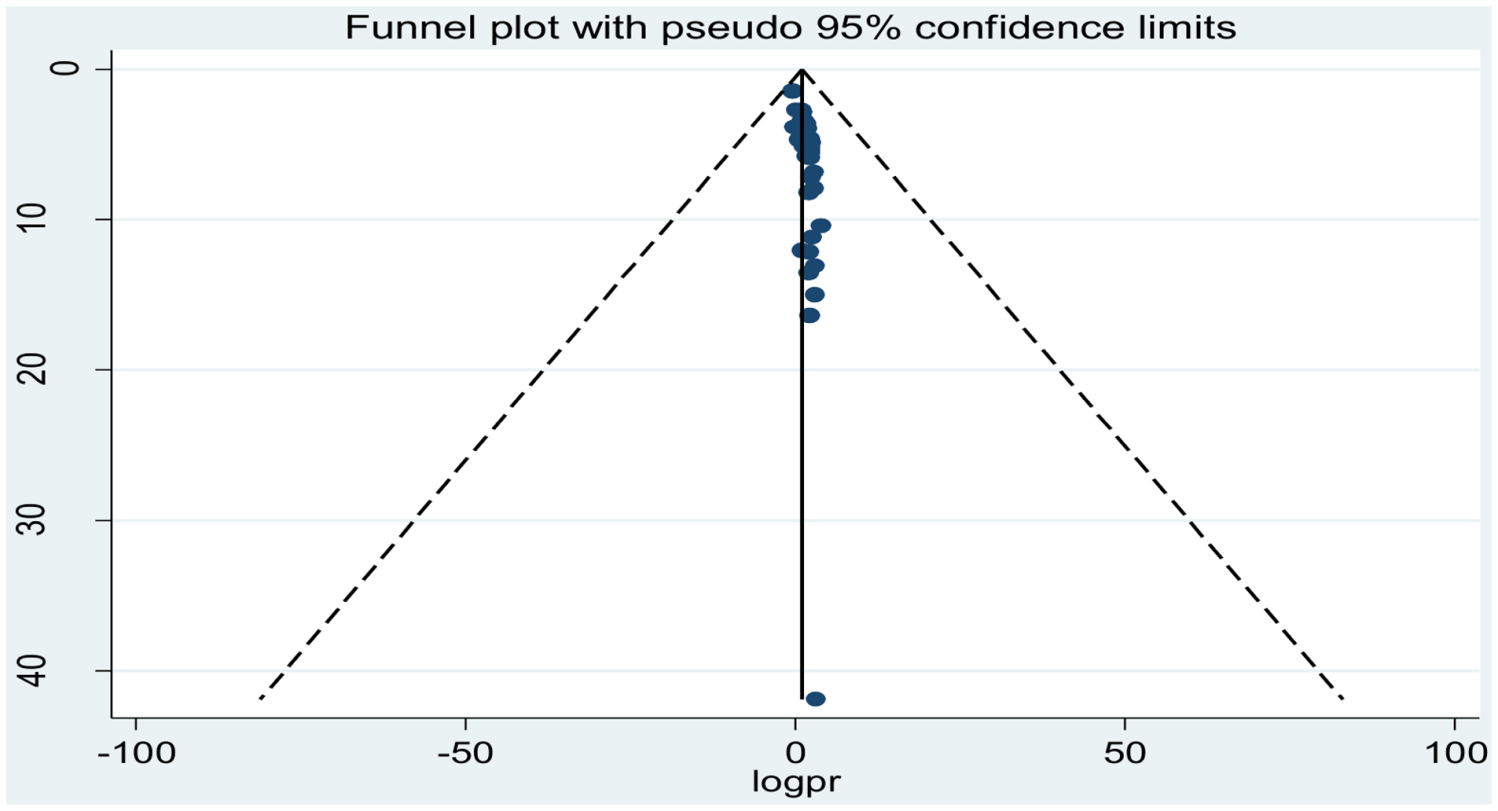
Funnel plot showing publication bias of studies reporting the pooled prevalence of tuberculosis among prisoners in sub-Saharan Africa, 2023.

To assess possible publication bias, a visually inspected funnel plot was used, which was statistically supported by Egger’s and Begg’s rank regression tests. The symmetrical distribution of the included studies in a large inverted funnel demonstrated the absence of a publication bias. With *p*-values of (*p* = 0.26) and (*p* = 0.15), respectively, the Egger and Begg rank tests revealed no publication bias among the included articles to estimate the pooled prevalence of tuberculosis among prisoners in sub-Saharan Africa (Figure 5).

### Sensitivity analysis

By excluding each study one at a time, a leave-out-one sensitivity analysis was used to determine the effect of a single study on the pooled prevalence of tuberculosis among prisoners in sub-Saharan Africa. According to the findings, no single study had a significant impact on the pooled estimate of tuberculosis among prisoners in sub-Saharan Africa (Table 3).

**Table 3:** Sensitivity analysis for the pooled prevalence of tuberculosis among prisoners in sub-Saharan Africa, 2023.

Study omitted	Estimate	[95% Confidence Interval]	
Abebe et al. (12)	3.9517572	2.6016049	5.3019094
Adane et al. (14)	3.9698453	2.610373	5.3293176
Adane et al. (13)	4.0264149	2.6838734	5.3689561
Addis et al. (15)	3.9512789	2.6003962	5.3021617
Adesokan et al. (16)	4.1032853	2.7440047	5.4625654
Agajie et al. (17)	3.9199085	2.5669565	5.2728605
Ali et al. (18)	3.9316785	2.5795114	5.2838459
Banda et al. (19)	4.9349384	3.4217978	6.4480796
Bayu et al. (20)	3.9951077	2.6426461	5.3475695
Berihun et al. (21)	3.9953275	2.6537507	5.3369045
Biyadgilign et al. (22)	3.9976826	2.6530216	5.3423433
Chekesa et al. (24)	3.8280344	2.484884	5.1711845
Chigbu and Iroegbu (23)	3.9933197	2.6507154	5.3359241
Dibissa et al. (25)	3.9965336	2.6471703	5.3458967
Fuge et al. (26)	4.0650678	2.7120979	5.4180374
Gebrecherkos et al. (27)	4.0001011	2.6476939	5.3525081
Gizachew et al. (28)	4.0395069	2.6816096	5.3974042
Habeenzu et al. (29)	4.018136	2.6775756	5.3586965
Henostriza et al. (30)	4.0268888	2.6752195	5.3785586
Jordan et al. (31)	4.074502	2.7083395	5.4406643
Kalonji et al. (32)	3.8945763	2.5479105	5.2412419
Kenyerere et al. (33)	4.0827537	2.722759	5.4427481
Keyomo et al. (35)	4.0945396	2.7121942	5.4768853
Kwabla et al. (36)	4.1072049	2.748898	5.4655113
Lawal et al. (36)	4.0595646	2.7042162	5.4149127
Maggard et al. (37)	4.0239921	2.6584303	5.3895535
Merid et al. (38)	3.9560311	2.6068742	5.3051877
Mmbaga et al. (41)	3.9964359	2.6378741	5.3549976
Moges et al. (40)	3.9682374	2.6221004	5.3143744
Mohammed et al. (39)	4.0767627	2.7016883	5.4518375
Mpeirwe et al. (42)	4.0155196	2.6738584	5.3571806
Noeske et al. (43)	4.0334573	2.6798909	5.3870234
Nyangulu et al. (44)	3.9854186	2.6217782	5.3490591
Owokuhaisa et al. (45)	4.2006702	2.8187807	5.5825596
Seri et al. (47)	3.9125924	2.5582464	5.2669387
Sesay et al. (46)	4.0129809	2.6708927	5.3550696
Telisinghe et al. (48)	4.0587621	2.6805737	5.4369502
Winsa et al. (49)	3.9790123	2.6369779	5.3210464
Zerdo et al. (50)	3.9120765	2.566848	5.2573047
Zishiri et al. (51)	3.9422443	2.5910163	5.2934723
Combined	4.0231154	2.6827336	5.3634972

### Trends of TB prevalence

**Figure 6:**
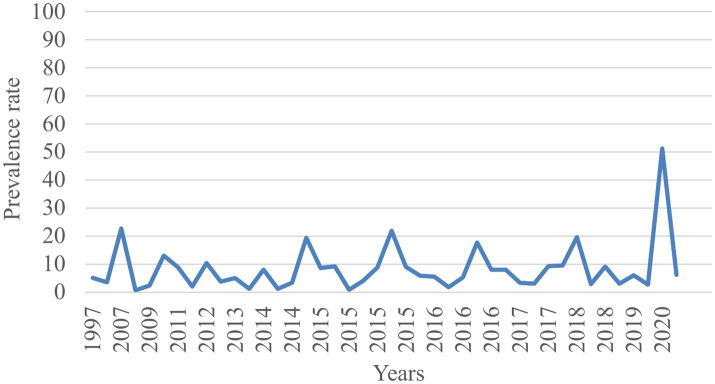
Time trend of TB prevalence among prisoners in sub-Saharan Africa from 1997 to 2020.

The trend analysis indicated that despite efforts to eradicate TB, the disease burden among prisoners in sub-Saharan Africa continued to rise from 1997 to 2020 (Figure 6).

## Discussion

There is evidence that the number of people developing tuberculosis is increasing in many low-and middle-income countries, and between 2019 and 2021, the number of deaths from tuberculosis also increased (1). In prisons, infectious diseases such as tuberculosis may spread more easily because segregation criteria are based on criminal characteristics rather than on public health concerns (52). As a result, the goal of this systematic review and meta-analysis was to report the most recent estimated pooled prevalence of tuberculosis among prisoners in sub-Saharan Africa.

The prevalence of tuberculosis among household contacts was 3.29% (95% CI: 2.35–4.23) (52). A recent systematic review has documented a 3- to 1,000-fold increase in the prevalence of TB in prisons compared to the general population (22). In SSA, it is estimated to be 6–30 times higher than that in the general population (53). According to the results of the current systematic review and meta-analysis, the pooled prevalence of tuberculosis among prisoners in sub-Saharan Africa was 4.02% (95% CI: 2.68–5.36). This finding is consistent with findings from Tajikistan 4.5% (54), South Africa 2.7% (51), and Ethiopia (4.0%) (55). This could be attributed to the similarity in tuberculosis diagnostic methods used in the incarcerated population.

However, the pooled prevalence of tuberculosis in this systematic review and meta-analysis was lower than that in an Ethiopian systematic review and meta-analysis (8.33%) (2). Furthermore, it was lower than that in studies conducted in Brazil 27.8% (56), Malaysia 7.7% (57), Nepal 10% (55), Iran 7.9% (58), South Africa (8.8%) (11), and Zambia (6.4%) (26). The lower prevalence found in this study could be attributed to differences in geographical location and the number of rooms with prisoners with poor ventilation.

The pooled prevalence of tuberculosis among prisoners in the current meta-analysis was higher than that in studies conducted in Brazil (1.89%) (59), Thailand (2.1%) (60), and Peru (2.5%) (61). The higher prevalence of tuberculosis in our study might be due to overcrowding and the difference in the incarcerated years of inmates.

Sub-group analysis of the pooled prevalence of tuberculosis among prisoners in sub-Saharan Africa showed no statistically significant difference (*p* = 0.188). Using diagnostic methods, tuberculosis was detected by Gene Xpert (4.97%), sputum smear microscopy (3.53%), and culture (2.88%). Xpert MTB/RIF’s suitability and feasibility as an MTB diagnostic method are attributed to its suitability and feasibility as a quick, reliable, controllable, simple, and cost-effective test (62). Gene Xpert uses DNA PCR technology to detect MTB and rifampicin resistance mutations simultaneously (63).

The sub-group analysis of this review also showed that the prevalence of tuberculosis among prisoners was higher in Ethiopia (7.10%) compared to other countries in sub-Saharan Africa. The variation in the prevalence of pulmonary TB within countries in prisons could be due to differences in diagnostic techniques, screening methods, overcrowding, and sociocultural and socioeconomic factors among the study participants.

An ongoing intervention for Tuberculosis (TB) in sub-Saharan Africa is the implementation of active case-finding and treatment programs within prisons. This involves screening all inmates for TB, providing treatment for those who test positive, and implementing infection control measures to prevent the spread of the disease within the prison environment. Additionally, TB preventive therapy is provided to high-risk inmates, such as those with HIV or other underlying health conditions, which helps to reduce the overall burden of TB within the prison population (64).

### Strengths and limitations of the study

The strength of this review is that it follows the recommended PRISMA guidelines. We also rigorously searched the literature in different databases and identified eligible studies. Moreover, in the present review, the heterogeneity among studies was low. While interpreting the results of this systematic review and meta-analysis, we considered the limitations of this review. We were forced to compare our findings with those of primary studies in some parts of the discussion because of a lack of adequate systematic reviews and meta-analyzes. The other limitation of this review is that we only considered articles written in the English language, which may result in the exclusion of other articles. Last but not least, we found studies conducted in 13 SSA countries, which may not represent prisoners throughout the whole region.

## Conclusion

The pooled prevalence of tuberculosis among prisoners in sub-Saharan Africa was prominently high based on this systematic review and meta-analysis. Therefore, to reach the end of the global TB epidemic, improvement in the prison setting is important. Screening on entry to the prison, periodical TB symptom screening, TB prevention training and information dissemination among the health staff in the prison and the inmates, and immediate treatment of diseased prisoners are important these measure to be put in place. Finally, this will help with the early identification and diagnosis of tuberculosis, which will reduce multidrug-resistant tuberculosis occurrence.

## Data availability statement

The raw data supporting the conclusions of this article will be made available by the authors, without undue reservation.

## Author contributions

The study was conceptualized and developed by YS and TM, who also conducted data analysis and interpretation and wrote the first draft. GA and YS built the search strategy, extracted the data, and assessed the quality of the studies included. The writing was reviewed and edited by YS and TM. All authors contributed to the article and approved the submitted version.
